# Non-coding RNA methylation modifications in hepatocellular carcinoma: interactions and potential implications

**DOI:** 10.1186/s12964-023-01357-0

**Published:** 2023-12-18

**Authors:** Qingmiao Shi, Qingfei Chu, Yifan Zeng, Xin Yuan, Jinzhi Wang, Yaqi Zhang, Chen Xue, Lanjuan Li

**Affiliations:** 1 State Key Laboratory for Diagnosis and Treatment of Infectious Diseases, National Clinical Research Center for Infectious Diseases, National Medical Center for Infectious Diseases, Collaborative Innovation Center for Diagnosis and Treatment of Infectious Diseases, The First Affiliated Hospital, Zhejiang University School of Medicine, Hangzhou, 310003 China; 2 Jinan Microecological Biomedicine Shandong Laboratory, Jinan, 250000 China

**Keywords:** HCC, ncRNA, m6A, m5C, m1A

## Abstract

RNA methylation modification plays a crucial role as an epigenetic regulator in the oncogenesis of hepatocellular carcinoma (HCC). Numerous studies have investigated the molecular mechanisms underlying the methylation of protein-coding RNAs in the progression of HCC. Beyond their impact on mRNA, methylation modifications also influence the biological functions of non-coding RNAs (ncRNAs). Here, we present an advanced and comprehensive overview of the interplay between methylation modifications and ncRNAs in HCC, with a specific focus on their potential implications for the tumor immune microenvironment. Moreover, we summarize promising therapeutic targets for HCC based on methylation-related proteins. In the future, a more profound investigation is warranted to elucidate the effects of ncRNA methylation modifications on HCC pathogenesis and devise valuable intervention strategies.

Video Abstract

**Supplementary Information:**

The online version contains supplementary material available at 10.1186/s12964-023-01357-0.

## Background

Hepatocellular carcinoma (HCC) ranks as the sixth most prevalent malignancy globally and one of the leading causes of cancer-related mortality [1, 2]. Projections indicate that by 2025, more than 1 million individuals will be diagnosed with liver cancer annually, with HCC accounting for over 90% of the cases [3]. The relative five-year survival rate for HCC is a mere 18%, underscoring its substantial burden on public health [4]. The incidence of HCC is influenced by many factors, such as geographic location, demographic attributes, and lifestyle choices [5]. Prominent among these factors are viral hepatitis and alcohol consumption, which increase the risk of HCC development [6]. Over the recent decades, the global incidence of HCC attributed to viral hepatitis has decreased owing to universal coverage of the hepatitis B virus (HBV) vaccine and increased accessibility to efficacious antiviral therapies for both HBV and hepatitis C virus (HCV) infections [7–9]. However, the prominence of nonalcoholic fatty liver disease and non-alcoholic steatohepatitis as culprits for HCC is gradually escalating, which is particularly evident within the United States [10, 11]. Early-stage HCC can be potentially cured by local ablation, surgical excision, and liver transplantation [12–14]. For medium-stage HCC, transarterial interventions such as transarterial chemoembolization (TACE) prove to be valuable therapeutic avenues [15]. For advanced HCC, receptor tyrosine kinase inhibitors and immunotherapy offer efficacious courses of treatment [16]. Despite strides in multidisciplinary treatment strategies, the prognosis for HCC remains suboptimal due to challenges such as early surgical recurrence and resistance to targeted therapies. Therefore, a critical imperative lies in unraveling the intricate molecular mechanisms underpinning HCC progression, aimed at identifying novel biomarkers and therapeutic targets.

Non-coding RNAs (ncRNAs) are a class of RNAs that do not participate in protein synthesis, accounting for over 90% of the RNA content in the human genome [17, 18]. Typically, ncRNAs are categorized based on their length, structure, and cellular localization. Among these, microRNAs (miRNAs), long ncRNAs (lncRNAs), and circular RNAs (circRNAs) represent three principal categories of ncRNAs, each with distinct biological functions. MiRNAs are a small fragment of RNA, approximately 20–25 nucleotide long, and can bind to complementary sequences on messenger RNA (mRNA), thereby degrading them through cleavage, destabilization, or inhibition of translation into proteins [19–22]. Conversely, both lncRNAs and circRNAs consist of more than 200 nucleotides. LncRNAs engage not only in epigenetic modifications and the cis or trans regulation of gene transcription but also participate in post-transcriptional regulation, affecting mRNA splicing, stability, and translation [23, 24]. CircRNAs, characterized by their covalently closed circular structure that enhances stability, serve as miRNA or protein sponges and regulate transcriptional variability as well as parental gene expression [25–27]. Over the past decade, the discovery of tens of thousands of ncRNAs has notably transformed researchers’ comprehension of gene expression regulation and disease progression. In particular, numerous studies of ncRNA biology have underscored their pivotal roles during HCC tumorigenesis, where they function as either oncogenic agents or tumor suppressors [28–30]. For instance, microRNA-93-5p has been identified as binding to the 3′-untranslated region (UTR) of mitogen-activated protein kinase kinase 2, thereby promoting HCC progression [31]. Another study revealed that circRNA-5692, acting as a miR-328-5p sponge, enhances DAB2IP expression, thereby inhibiting HCC growth [32]. Additionally, the hypoxia-induced lncRNA DACT3-AS1 has been found to facilitate the interaction between HDAC2 and FOXA3, thereby upregulating PKM2 expression and promoting the metastasis of HCC [33].

There is a burgeoning interest in elucidating the role of epigenetic regulation in the pathogenesis of HCC, with a particular focus on RNA methylation modifications. RNA methylation refers to the chemical modification phenomenon wherein methyl groups are selectively added to the methyl adenine of RNA under the catalysis of methyltransferases [34, 35]. This modification has been observed across various RNA categories, including mRNA, transfer RNA (tRNA), ribosomal RNA, small nuclear RNA, miRNA, lncRNA, and circRNA [36–38]. Among the most prevalent RNA methylation modifications in mammals are N6-methyladenosine (m6A), N1-methyladenosine (m1A), 5-methylcytosine (m5C), pseudouridine (Ψ), and 7-methylguanine (m7G) [39–42]. At the post-transcriptional level, RNA methylation dynamically and reversibly regulates RNA stability, localization, transport, cleavage, and translation [43–45]. This intricate control is executed by a trio of functional methylation-related proteins: methyltransferases (“writers”), demethylases (“erasers”), and methylated reading proteins (“readers”). In recent years, the role of RNA methylation modifications in the oncogenesis of HCC has been progressively garnering attention. For instance, one study reported that methyltransferase-like 3 (METTL3) mediates m6A modification of FOXO3 mRNA, thereby influencing the drug tolerance of HCC to sorafenib [46]. Another study revealed that the methylated reading protein Aly/REF export factor (ALYREF) regulates m5C methylation levels in target RNA, consequently facilitating the progression of liver cancer [47]. Moreover, the m1A methyltransferase complex hnRNPK/TRMT61A has been shown to augment m1A methylation of tRNA, thereby regulating cholesterol metabolism in liver cancer stem cells [48]. This mechanism, in turn, propels liver tumorigenesis through the Hedgehog signaling pathway. Thus, the development of novel drugs targeting methylation-related proteins may provide novel avenues for the therapy of HCC.

Numerous studies have delved into the mechanisms underlying the methylation of protein-coding RNAs in HCC. In addition to mRNA, methylation modifications also affect the metabolism and functioning of ncRNAs, thereby playing a role in the proliferation, invasion, and drug resistance in HCC cells. In this review, we summarize the interactions between methylation modifications and ncRNAs in HCC, highlighting recent advancements in this burgeoning field of research. This review focuses on the potential implications of m6A and m5C methylation modifications in miRNAs, lncRNAs, and circRNAs. Additionally, it probes into the regulatory mechanisms exerted by miRNAs, lncRNAs, and circRNAs on m6A and m5C methylation-related proteins. Ultimately, we discuss the present status and the future outlook for targeting RNA methylation-related proteins in the treatment of HCC.

## Primary ncRNA methylation modifications in HCC

Common RNA methylation modifications, including m6A, m5C, m1A, and Ψ, have been identified in ncRNAs. Here, we primarily summarize the m6A, m5C and m1A methylation modifications in ncRNAs.

### m6A methylation of ncRNAs

**Fig. 1:**
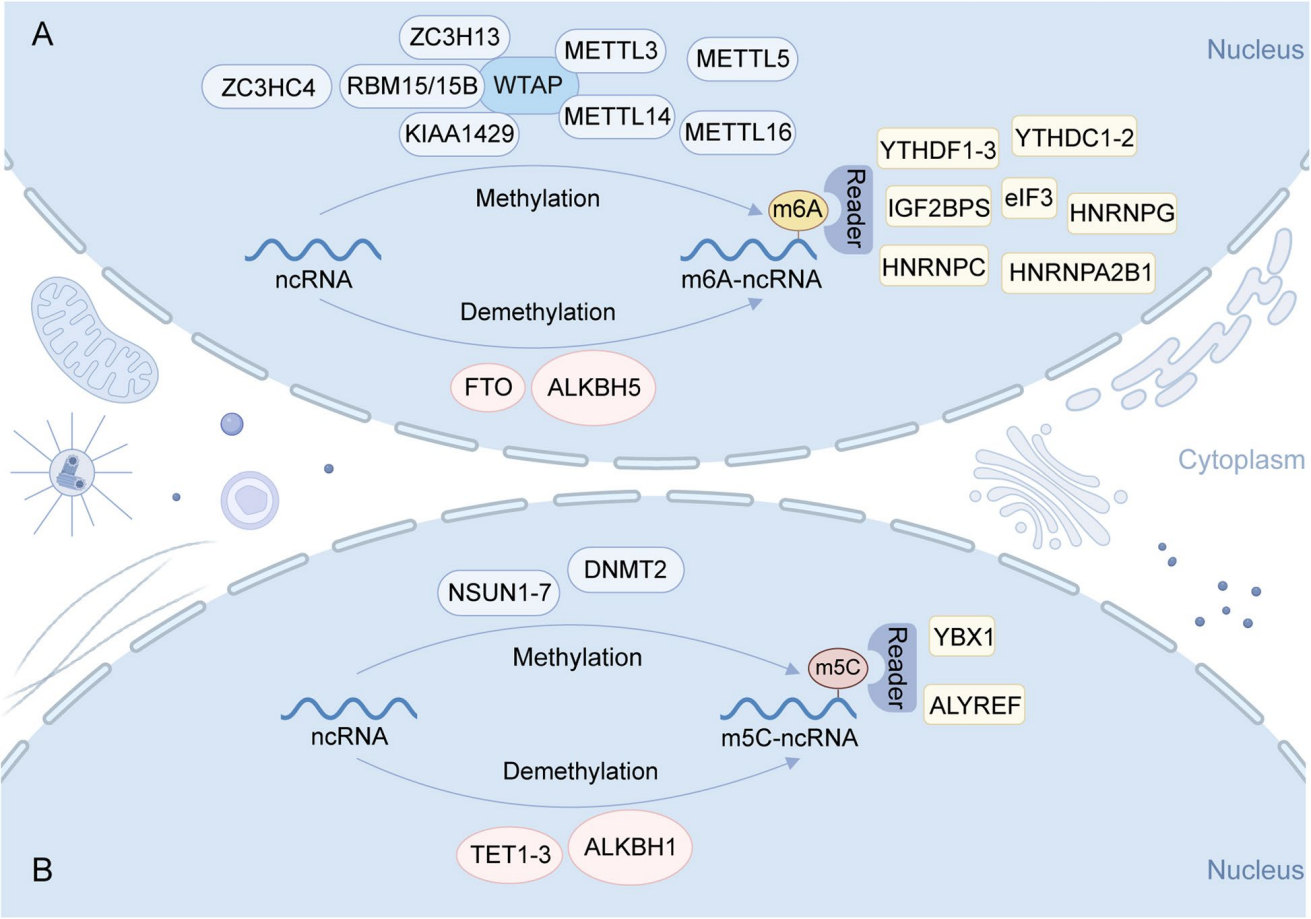
Overview of the m6A and m5C methylation modification in ncRNA. **A** m6A methylation was catalyzed by methyltransferase enzymes, including METTL3, METTL14, METTL5, METTL16, ZC3H13, ZCCHC4, WTAP, KIAA1429, and RBM15/15B. FTO and ALKBH5 participate in the demethylation process. Specific “reader” protein, such as YTHDF1-3, IGF2BP1-2, HNRNPA2B1, hnRNPC, bind to and recognize these methylation sites. **B** The enzymatic catalysis of m5C methylation involves primarily the NSUN1–NSUN7 and DNMT2. The TET1-TET3 and ALKBH1 can facilitate m5C demethylation. ALYREF and YBX1 are two “reader” proteins for m5C-modified ncRNA. Created with BioRender.com

The m6A methylation was first reported in 1974 [49–51]. It involves the methylation of the sixth nitrogen atom on the RNA molecule, catalyzed by methyltransferase enzymes. Subsequently, specific “reader” proteins bind to and recognize these methylation sites. Demethylation of these sites is achieved through the actions of demethylase enzymes. Currently known m6A methyltransferases include METTL3, METTL14, METTL5, METTL16, zinc finger CCCH-type containing 13 (ZC3H13), zinc finger CCHC domain-containing protein 4 (ZCCHC4), Wilms tumor 1-associated protein (WTAP), KIAA1429, and RBM15/15B [52, 53]. Notably, fat mass and obesity-associated protein (FTO) and alkylation repair homolog protein 5 (ALKBH5) are pivotal RNA demethylases [54, 55]. “Reader” proteins primarily include YTH N6-methyladenine RNA binding protein 1 (YTHDF1), YTHDF2, YTHDF3, insulin-like growth factor 2 mRNA-binding protein 1 (IGF2BP1), IGF2BP2, heterogeneous nuclear ribonucleoprotein A2/B1 (HNRNPA2B1), and heterogeneous nuclear ribonucleoprotein C (hnRNPC) (Fig. 1A) [56].

The m6A modification not only regulates the shear, transport, stability, and degradation of ncRNAs but also modulates cellular functions by altering their expression [57]. This modification can affect the maturation of miRNAs. In the case of lncRNAs, m6A modification may regulate the function of lncRNAs by regulating the structure of local lncRNAs, thereby inducing the binding of RNA-binding proteins [58]. In addition, m6A modifications might play a role in shaping the interplay between RNA and DNA, particularly at specific DNA sites, by influencing the triple helix structure of lncRNAs. Moreover, m6A can promote the export of circRNAs to the cytoplasm, driving circRNA translation and mediating circRNA degradation [59–61].

### m5C methylation of ncRNAs

In the 1970s, researchers discovered m5C modifications, wherein “m5C” signifies the addition of a methyl group to the fifth carbon atom of the cytosine base in RNA [51, 62]. This m5C methylation modification is prevalent in both mRNA and ncRNA. The enzymatic catalysis of m5C methylation involves primarily the NOL1/NOP2/SUN domain (NSUN) family (NSUN1–NSUN7) and DNA methyltransferase 2 (DNMT2) [63]. Acting as “erasers” to facilitate m5C demethylation in RNA, the ten-eleven translocation (TET) family (TET1-TET3) and ALKBH1 play crucial roles [62]. The functional significance of RNA modification predominantly relies on “readers.“ In this context, ALYREF and Y-box binding protein 1 (YBX1) are two recognition proteins for m5C-modified mRNA (Fig. 1B). These proteins exert biological effects by identifying and binding to m5C sites.

As a reversible epigenetic modification, the m5C modification of ncRNA affects the molecular trajectory of the modified ncRNA, playing a vital role in multiple biological processes, including ncRNA stability regulation, protein binding, and transcriptional regulation [64]. For example, the methyltransferase NSUN2 can interact with the lncRNA NKILA, thereby increasing its m5C levels and facilitating its interaction with YBX1 [65]. In addition, a previous study uncovered the substantial presence of m5C sites on circRNAs in human HCC tissues. The distinctive distribution pattern of m5C modifications on circRNA in HCC exhibited correlations with specific metabolic pathways [66]. Therefore, the exploration of m5C methylation of ncRNA assumes considerable significance, offering insights into the underlying mechanisms of disease pathogenesis and progression.

### m1A methylation of ncRNAs

The m1A methylation, a critical internal RNA modification that has gradually received attention from researchers since it was first reported in 1961 [67], occurs on the first nitrogen atom of adenosine in RNA molecule. m1A has been determined in diversified RNA types, including tRNA, rRNA, mRNA, and lncRNA [40]. Similar to the dynamic modification of m6A and m5C, m1A is also regulated by functional methylation-related proteins called “writers”, “erasers”, and “readers”. The “writers” mainly includes tRNA methyltransferase 6 (TRMT6), TRMT61A, TRMT61B, TRMT10C, and nucleomethylin (NML) [68, 69]. ALKBH1, ALKBH3, ALKBH7, and FTO compose the “erasers” of m1A [70–73]. The “readers”, namely YTHDF1/2/3 and YTHDC1, are responsible for recognizing and binding to the m1A site [74].

m1A modification is considered to participate in the pre-RNA processing, regulate the structure and stability of RNA [72, 75]. Additionally, recent studies have shown that m1A affect the process of translation via modifications in tRNA [70], rRNA, and mRNA. By function, Wu et al. revealed that m1A demethylase ALKBH3 could regulate the glycose metabolism of tumor cells in a demethylation activity-dependent pattern [76]. Specifically, the m1A regulates the translation of ATP5D through YTHDF1/eRF3, and the m1A negatively regulates the mRNA stability of E2F1, thereby initiating the transcription of ATP5D. Another study demonstrated that TRMT61A mediates the m1A modification of tRNA, accelerates the translation of various key proteins after activation of CD4^+^T cells, and ensures the rapid immune response of CD4^+^T cells [77]. These studies broaden the understanding of the biological behavior of m1A-regulated RNA, and provide a theoretical basis for the development of novel tumor intervention strategies based on m1A modification. However, the study of biological function of m1A modification is still in its infancy. The interaction between m1A modification and ncRNA in HCC biology need to be further studied.

## Biological function of methylation modification in ncRNAs in HCC

A growing body of evidence suggests that the methylation of ncRNAs plays a crucial role in the development of HCC. Here, we mainly summarize the biological functions associated with m6A and m5C methylation modifications of miRNAs, lncRNAs, and circRNAs in HCC (Table 1).

**Table 1:** Biological functions associated with methylation modifications of ncRNAs in HCC

Methylation type	Category	Methylation-related proteins	Function of ncRNA methylation modification	Target ncRNA	Expression of ncRNA (tumor vs. normal)	Effects of ncRNA	Function of ncRNA	Molecular mechanism	Refs
m6A	Writer	METTL14	METTL14 regulates pri-miR126	MicroRNA-126	Downregulated	Anti-oncogene	Inhibits tumor metastasis	METTL14-DGCR8-pri-miR126 axis	[78]
m5C	Reader	YBX1	YBX1 suppresses miR-205/200b maturation	MiR-205/200b	/	/	/	miR-205/200b-ZEB1 axis	[79]
m5C	Eraser	TET1	TET1 activates miR-34a by demethylating miR-34a	MicroRNA-34a	Downregulated	Anti-oncogene	Inhibits proliferation, migration, invasion, tumorigenesis, metastasis, and inflammation, and promotes cell autophagy and apoptosis	miR-34a/BACH1/p53 axis	[80]
m6A	Writer	METTL3	METTL3 regulates the m6A modification of MEG3 and its expression	LncRNA MEG3	Downregulated	Anti-oncogene	Inhibits proliferation, migration, and invasion	MEG3/miR-544b/BTG2 axis	[81]
m6A	Writer	METTL3	METTL3-mediated m6A modification leads to LINC00958 upregulation by stabilizing its RNA transcript	LINC00958	Upregulated	Oncogene	Facilitates lipogenesis and progression	METTL3/LINC00958/miR-3619-5p/HDGF axis	[82]
m6A	Writer	METTL3	METTL3 maintains lnc-CTHCC stability and increases its expression, and is recognized by IGF2BP1/IGF2BP3	lnc-CTHCC	Upregulated	Oncogene	Promotes growth and metastasis	METTL3–IGF2BP1/IGF2BP3–lnc-CTHCChnRNP K–YAP axis	[83]
m6A	Writer/reader	METTL3, YTHDF2	METTL3 increases LINC01273 m6A modification, which is followed by LINC01273 decay in the presence of YTHDF2	LINC01273	Upregulated	Oncogene	Confers sorafenib resistance	LINC01273/miR-600/METTL3 axis	[84]
m6A	Writer/reader	METTL14/IGF2BP2	METTL14 serves as a m6A writer of ARHGAP5-AS1, and IGF2BP2 stabilizes the lncRNA as its m6A reader	LncRNA ARHGAP5-AS1	Upregulated	Oncogene	Promotes malignant behavior of HCC cells	METTL14-IGF2BP2/ARHGAP5-AS1/CSDE1-TRIM28/VIM-RAC1/ERK pathway	[85]
m6A	Writer	METTL16	METTL16 decreases the stability of RAB11B-AS1 transcript	LncRNA RAB11B-AS1	Downregulated	Anti-oncogene	Represses proliferation, migration, and invasion of HCC cells, promotes apoptosis in HCC cells, and inhibits HCC tumoral growth in vivo	METTL16–RAB11B-AS1 regulation axis	[86]
m6A	Writer	ZCCHC4	ZCCHC4 interacts with AL133467.2	LncRNA AL133467.2	/	/	Promotes chemoresistance of HCC cells to DNA-damaging agent (DDA), and inhibits DNA-damage-induced apoptosis in HCC cells	ZCCHC4-AL133467.2-γH2AX complex	[87]
m5C	Writer	NSUN2	NSUN2-mediated RNA methylation possibly stabilize H19 lncRNA	LncRNA H19	Upregulated	Oncogene	Promote the occurrence and development of HCC	NSUN2/lncRNA H19/G3BP1 axis	[88]
m6A	Reader	YTHDF2	YTHDF2 possibly decreases the expression levels of lncAY in HCC in an m6A-dependent manner	LncAY	Upregulated	Oncogene	Accelerates HCC cell proliferation and migration	lncAY/YTHDF2/BMI1/Wnt/β-catenin axis	[89]
m6A	Reader	YTHDF2	Increases the splicing of lncFAL	LncFAL	Upregulated	Oncogene	lncFAL reduces susceptibility to ferroptosis	YTHDF2/lncFAL/HDLBP/FSP-dependent anti-ferroptosis mechanism	[90]
m6A	Reader	IGF2BP1	IGF2BP1 specifically regulates HULC expression and stability	LncRNA HULC	Upregulated	Oncogene	HULC exhibits correlations with staging and grading in HCC	IGF2BP1/lncRNA HULC/CNOT1 axis	[91]
m6A	Eraser	ALKBH5	ALKBH5 upregulates NEAT1 expression by inhibiting m6A enrichment	LncRNA NEAT1	Upregulated	oncogene	Promotes proliferation and migration and inhibits apoptosis	ALKBH5/NEAT1/miR-214 axis	[92]
m6A	Eraser	ALKBH5	ALKBH5 increases the stability of LINC01468 and upregulate its expression	LINC01468	Upregulated	Oncogene	Promotes proliferation	ALKBH5/LINC01468/SHIP2 axis	[93]
m6A	Writer/reader	METTL3, YTHDC1	METTL3 mediates the degree of methylation modification of hsa_circ_0058493, and YTHDC1 binds to hsa_circ_0058493 and promote its translocation from the nucleus to the cytoplasm	Hsa_circ_0058493	Upregulated	Oncogene	Promotes growth and metastasis	METTL3-hsa_circ_0058493-YTHDC1 axis	[94]
m6A	Writer/reader	METTL3, YTHDC1	METTL3 directs the formation of circHPS5, and YTHDC1 facilitates the cytoplasmic output of circHPS5 under m6A modification	CircHPS5	Upregulated	Oncogene	Promotes EMT and cancer stem-like cell (CSC) phenotypes	METTL3-YTHDC1/circHPS5/miR-370/HMGA2 regulatory model	[95]
m6A	Writer/reader	METTL3, YTHDC1	METTL3 increases the m6A modification of circ-ARL3, and YTHDC1 promotes its reverse splicing and biogenesis	Circ-ARL3	Upregulated	Oncogene	Promotes proliferation and invasion	circ-ARL3/miR-1305 axis	[96]
m6A	Writer	METTL3	METTL3 knockdown partially counteracts hsa_circ_0008583 overexpression-mediated influence on HCC cell behavior	Has_circ_0008583	Upregulated	Oncogene	Promotes proliferation, migration, and invasion	hsa_circ_0008583/miR-1301-3p/METTL3 axis	[97]
m6A	Writer	KIAA1429	Regulates the expression of circDLC1	CircDLC1	Downregulated	Anti-oncogene	Inhibits the proliferation and metastasis of hepatoma cells	KIAA1429/circDLC1/HuR/MMP1 axis	[98]
m6A	Eraser/writer	ALKBH5, METTL3	ALKBH5 and METTL3 bind and regulate m6A-modified circ-CCT3	Circ-CCT3	Upregulated	Oncogene	Promotes proliferation, invasion, and migration	circ-CCT3/miR-378a-3p/FLT1 axis	[99]
m6A	Eraser/reader	ALKBH5, YTHDF2, YAP1	circCPSF6 is dominated by ALKBH5-mediated demethylation, which is followed by recognition and destabilization by YTHDF2	CircCPSF6	Upregulated	Oncogene	Maintains cell proliferation and tumorigenicity and reinforces cell motility and tumor metastasis	ALKBH5-YTHDF2/circCPSF6/PCBP2/YAP1	[100]

### Biological function of methylation modifications in miRNAs

METTL14, a pivotal active component of the m6A methyltransferase complex, primarily localizes within the nucleus. Studies have demonstrated a notable downregulation of METTL14 expression in liver cancer tissues [101, 102]. Depletion of METTL14 has been shown to bolster the metastatic capacity of HCC cells, correlating with unfavorable overall survival rates among patients with HCC. MiR-126 is a tumor suppressor and plays a vital role in tumor metastasis. Research has shown that miR-126 functions as a downstream effector of METTL14 [78]. Moreover, overexpression of METTL14 facilitates its interaction with the microprocessor protein DGCR8, thereby enhancing the transformation of primary miR-126 into mature miRNA through an m6A-dependent mechanism, ultimately inhibiting HCC metastasis.

YBX1, alternatively referred to as YB1, serves as a “reader” protein for m5C modifications. YB1 is a carcinogen, and its elevated expression is closely linked to a dismal prognosis for HCC [103]. Liu et al. found that YB1 impedes the maturation of miR-205 and miR-200b by interacting with DGCR8, DICER, TUT4, and TUT1 [79]. Consequently, this engagement accentuates the expression of ZEB1, thereby facilitating the migration and invasion of HCC.

TET1, an “eraser” enzyme for m5C modifications, exhibits diminished expression within HCC tissues [104]. The absence of TET1 has been correlated with an unfavorable prognosis in HCC. The upregulation of TET1 inhibits the proliferation, migration, and invasion of HCC cells. Sun et al. found that TET1 activates miR-34a through demethylation of the 272/380 bp region within the miR-34a promoter. BACH1, a downstream target of miR-34a, mediates the P53 signaling pathway [80]. This intricate mechanism underscores how TET1 curtails HCC tumor genesis and metastasis through modulation of the miR-34a/BACH1/p53 axis, consequently fostering autophagy and apoptosis.

### Biological function of methylation modifications in lncRNAs

**Fig. 2:**
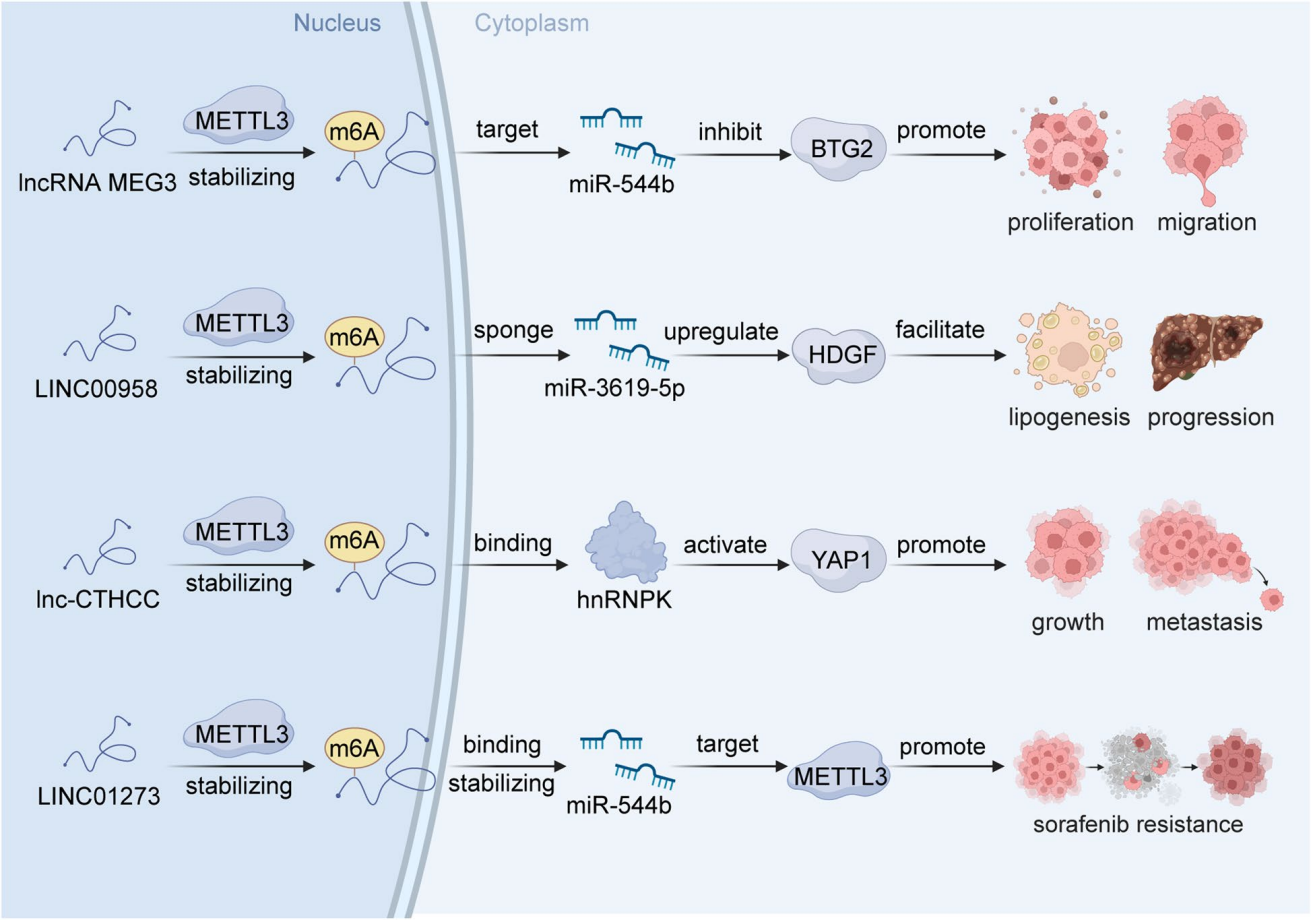
The molecular mechanism of METTL3 mediated m6A modifications in lncRNAs. METTL3 mediated m6A modifications could stabilize lncRNAs, such as lncRNA MEG3, LINC00958, LCC-CTHCC, and LINC01273, followed by regulate the downstream effectors, participating in multiple biological processes of HCC, including proliferation, migration, lipogenesis, metastasis, sorafenib resistance. Created with BioRender.com

METTL3 is the core protein of the m6A methyltransferase complex and plays a key role in m6A modifications of lncRNAs (Fig. 2) [105]. As elucidated by Wu et al., METTL3 mediates m6A modification of the lncRNA MEG3, subsequently leading to the downregulation of MEG3 [81]. This in turn exerts a notable influence on the malignant behavior of HCC through the MEG3/miR-544b/BTG2 axis. As established by a previous study, the lncRNA LINC00958 is associated with lipogenesis and is overexpressed in HCC. Moreover, the upregulation of LINC00958 is associated with an unfavorable overall survival prognosis. The m6A modification catalyzed by METTL3 leads to the upregulation of LINC00958 expression by reinforcing its transcriptional stability [82]. Consequently, LINC00958 operates as a molecular sponge for miR-3619-5p, culminating in the upregulation of hepatoma-derived growth factor (HDGF). This signaling cascade contributes to adipogenesis and the progression of HCC. Lnc-CTHCC, a conserved cancer-testis lncRNA, is highly expressed in testis and HCC. Xia et al. showed that METTL3-mediated m6A modifications in LCC-CTHCC are recognized by IGF2BP1 and IGF2BP3, crucially stabilizing LCC-CTHCC and increasing its expression in HCC [83]. Lnc-CTHCC can directly bind hnRNPK, thereby activating YAP1 transcription and, in turn, fostering the growth and metastasis of HCC. Additionally, both METTL3 and YTHDF2 mediate m6A modifications of LINC01273. Furthermore, LINC01273 increases sorafenib resistance in HCC by regulating the miR-600/METTL3 axis [84]. Targeting the LINC01273/miR-600/METTL3 signaling pathway emerges as a potential novel therapeutic strategy for effectively managing patients with sorafenib-resistant HCC.

METTL14, METTL16, and ZCCHC4 are crucial m6A methyltransferases. IGF2BP2 primarily functions as the “reader” protein. LncRNA ARHGAP5-AS1 is significantly overexpressed in HCC, with its m6A modification orchestrated by METTL14 and IGF2BP2, enhancing its stability. Liu et al. [85]. revealed that ARHGAP5-AS1 interacts with the oncoprotein CSDE1, orchestrating the regulation of carcinogenic RNA and driving the malignant tendencies of HCC. METTL16 is overexpressed in HCC, a factor that contributes to HCC tumorigenesis. METTL16 mediates the m6A modification of RAB11B-AS1, consequently diminishing the stability of its transcript and leading to the downregulation of RAB11B-AS1 expression [86]. Functioning as an RNA-binding protein, ZCCHC4 exhibits abnormal upregulation in HCC and is associated with a poor prognosis. In vitro and in vivo experiments have shown that ZCCHC4 enhances the chemotherapy resistance in HCC cells against DNA-damaging agents. Moreover, its interaction with the lncRNA AL133467.2 impedes apoptosis induced by DNA damage [87]. NSUN2, an m5C RNA methyltransferase, participates in the regulation of cell proliferation and metastasis and is upregulated in various tumors [42]. Sun et al. found that NSUN2 mediated m5C modification of lncRNA H19, thereby augmenting the stability of this lncRNA [88]. Through specific binding to the oncoprotein G3BP1, the m5C-modified lncRNA H19 triggers the accumulation of MYC, subsequently fostering poor differentiation of HCC.

Distinct RNA-binding proteins, known as “reader” proteins, play pivotal roles in executing specific biological functions of methylated RNA. The primary functions of these “reader” proteins primarily include the specific binding to the m6A methylation region, attenuation of homologous RNA-binding protein interactions, and modulation of RNA secondary structures to influence protein–RNA interactions. As elucidated by Chen et al., YTHDF2 orchestrates the m6A modification of lncAY, leading to the upregulation of BMI1 expression in HCC [89]. This, in turn, triggers activation of the Wnt/β-catenin signaling pathway, thereby contributing to HCC progression. YTHDF2 further engages in m6A-dependent splicing of lncFAL [90]. The resultant lncFAL then engages with ferroptosis suppressant protein 1 (FSP1), competitively thwarting TRIM69-mediated polyubiquitination degradation of FSP1 and thereby diminishing ferroptosis susceptibility. Moreover, IGF2BP1 specifically binds to the HCC-associated lncRNA HULC and plays a crucial role in RNA metabolism regulation [91]. Mechanistically, IGF2BP1 recruits the CCR4-NOT deadenylase complex, subsequently instigating the degradation of lncRNA HULC.

ALKBH5, a key demethylase of m6A, is downregulated in HCC. Chen et al. validated that ALKBH5 is a tumor suppressor factor in HCC, with its diminished expression correlating with poor overall survival in patients with HCC [106]. Ahati et al. further demonstrated ALKBH5’s ability to curtail m6A enrichment, resulting in the upregulation of the lncRNA NEAT1’s expression [92]. This ALKBH5-mediated lncRNA NEAT1 then acts as a sponge for miR-214, promoting the proliferation and migration of HCC cells. Moreover, m6A-modified LINC01468 is dependent on ALKBH5, which can enhance the stability of LINC01468 and upregulate its expression [93]. Upregulated LINC01468, in turn, interacts with SHIP2, enhancing CUL4A-associated degradation and enabling the activation of the PI3K/AKT/mTOR signaling pathway, thereby fueling lipogenesis and HCC progression.

### Biological function of methylation modification in circRNAs

CircRNAs are a distinctive form of closed circular ncRNAs, primarily produced by variable shear processing of pre-Mrna [107]. Notable for their stability and conservation, circRNAs have garnered increasing attention owing to their potential significance in diverse biological contexts. In the progression of HCC, accumulating evidence underscores the pivotal role of circRNA methylation [108–110]. Wu et al. revealed that hsa_circ_0058493 is highly expressed in HCC and positively correlates to a poor prognosis [94]. Moreover, METTL3 mediates the m6A methylation level of hsa_circ_0058493, with YTHDC1 facilitating its translocation from the nucleus to the cytoplasm, thereby modulating intracellular localization. Mechanistically, m6A-modified hsa_circ_0058493 was demonstrated to promote HCC development through the METTL3/hsa_circ_0058493/YTHDC1 axis. In addition, m6A-modified circHPS5HCC is highly expressed in HCC tissues, contributing to epithelial–mesenchymal transition (EMT) and the induction of cancer stem-like cell phenotypes. Mechanistically, METTL3 mediates circHPS5 generation, and YTHDC1 accelerates circHPS5 output from the cytoplasm [95]. Acting as a miR-370 sponge, circHPS5 influences the expression of HMGA2, thereby promoting the tumorigenesis of HCC. circ-ARL3, an HBV-associated circRNA, promotes malignant phenotypes in HBV-associated HCC. METTL3 enhances the m6A modification degree of circ-ARL3, and the binding of YTHDC1 and circ-ARL3 accelerates reverse splicing and biogenesis of circ-ARL3 [96]. Functioning as a miR-1305 sponge, circ-ARL3 further accentuates HBV-associated HCC tumor formation. Moreover, METTL3 mediates the effects of hsa_circ_0008583 on the behavior of HCC cells [97]. Furthermore, hsa_circ_0008583 accelerates the development of HCC via the miR-1301-3p/METTL3 axis.

KIAA1429 is a crucial component of the m6A methyltransferase complex and plays a vital role in m6A modification [111]. Elevated expression of KIAA1429 in HCC tissues was observed, correlating with poorer prognoses. KIAA1429 was found to enhance the development of HCC through m6A-dependent GATA3 post-transcriptional modification [112]. Additionally, KIAA1429 regulates the expression of circDLC1. Further studies have shown that circDLC1 interacts with the RNA-binding protein HuR interactions, thereby inhibiting the interplay between HuR and MMP1 mRNAs and subsequently curtailing HCC proliferation and motility [98]. Except for KIAA1429, Liu et al. revealed the involvement of ALKBH5 and METTL3 in binding to circ-CCT3, mediating its m6A modification. Upregulated in HCC, circ-CCT3 was proposed to function as a miR-378-3p sponge, thereby elevating FLT-1 expression and intensifying HCC proliferation, invasion, and migration [99]. Another study highlighted ALKBH5’s role in circCPSF6 demethylation, with YTHDF2 recognizing and destabilizing it [100]. M6A-modified circCPSF6 was shown to competitively bind with PCBP2, attenuating its interaction with YAP1 mRNA, thus activating YAP1 and sustaining tumorigenicity and metastasis of HCC.

## NcRNAs regulate methylation-related proteins in HCC

Both m6A and m5C modifications play pivotal roles in mediating the methylation modification of ncRNA. In turn, ncRNAs also modulate the expression levels of methylation-related proteins, thus affecting the occurrence and development of HCC. Here, we generalize the recent advancements pertaining to the regulatory effects of miRNAs, lncRNAs, and circRNAs on methylation-related proteins in HCC (Table 2).

**Table 2:** ncRNA regulating methylation-related proteins in HCC

NcRNA	Function of ncRNA	Methylation type	Category	Target methylation-related proteins	Expression of proteins (tumor v proteins s. normal)	Prognosis associated with overexpressed proteins	Function of methylation-related proteins	Molecular mechanism	Refs
miR-139-5p	miR-139-5p negatively regulates WTAP expression	m6A	Writer	WTAP	Upregulated	Poor	Promotes proliferation and invasion	miR-139-5p/WTAP axis	[113]
miR-1275	miR-1275 regulates IGF2BPs expression	m6A	Reader	IGF2BPS	/	/	/	miR-1275/IGF2BPs axis	[114]
miR-1275	miR-1275 regulates IGF2BPs expression	m6A	Reader	IGF2BPS	Upregulated	/	/	miR-1275/IGF2BPs axis	[115]
miR-188-5p	miR-188-5p regulates hnRNPA2B1 expression	m6A	Reader	HNRNPA2B1	Upregulated	Poor	/	miR-188-5p/hnRNPA2B1/PKM2 pathway	[116]
miR-4666a-5p和miR-6124	miR-4666a-5p and miR-6124 bind within the 3′-UTRs of ALYREF	m5C	Reader	ALYREF	Upregulated	Poor	/	/	[117]
miR-3190-5p	miR-3190 targets the 3′UTR of ALKBH5	m6A	Eraser	ALKBH5	Downregulated	Favorable	Suppresses formation and progression	EV-miR-3190/ALKBH5 axis	[118]
miR-22-3p	miR-22-3p directly targets TET2	m5C	Eraser	TET2	Downregulated	Favorable	Suppresses HCC stemness and metastasis	β-catenin/miR-22-3p/TET2 regulatory axis	[119]
miR-29	miR-29b targets TET1 and modulates TET1 expression	m5C	Eraser	TET1	Downregulated	Favorable	Represses cell proliferation	feedback of TET1-miR-29 family	[120]
MiR-520b	miR-520b targets 3′UTR of TET1 mrna	m5C	Eraser	TET1	/	/	/	miR-520b/TET1 axis	[121]
LINC00839	LINC00839 sponges miR-144-3p and regulates the expression of WTAP	m6A	Writer	WTAP	/	/	/	LINC00839/miR-144-3p/WTAP axis	[122]
lncRNA ILF3-AS1	ILF3-AS1 increases ILF3 m6A level by recruiting METTL3	m6A	Writer	METTL3	/	/	/	ILF3-AS1-METTL3ILF3 signaling axis	[123]
lncRNA miR503HG	miR503HG interacts with HNRNPA2B1 and mediates ubiquitination and degradation of HNRNPA2B1	m6A	Reader	HNRNPA2B1	Upregulated	Poor	/	miR503HG/HNRNPA2B1/NF-κB pathway	[124]
LncRNA-uc002mbe.2	uc002mbe.2 interacts with hnRNPA2B1	m6A	Reader	HNRNPA2B1	/	/	/	uc002mbe.2/hnRNPA2B1/AKT/p21 axis	[125]
Linc01612	Linc01612 interacts with YBX1 and promotes the ubiquitin-mediated degradation of YBX1	m5C	Reader	YBX1	Upregulated	Poor	/	Linc01612/YBX1/miR-494/ATF3/p53	[126]
Long noncoding RNA AWPPH	IncRNA-AWPPH promotes YBX1-mediated activation of PIK3CA transcription and activates the PI3K/AKT pathway	m5C	Reader	YBX1	/	/	Promotes tumor growth and metastasis	lncRNA-AWPPH/YBX1/PIK3CA/PI3K/AKT axis	[127]
circRERE	circRERE regulates the expression of ZC3H13	m6A	Writer	ZC3H13	Downregulated	Favorable	Inhibits HCC cell viability, promotes apoptosis, and reduces invasion.	circRERE/miR-1299/ZC3H13/GBX2 axis	[128]
rtcisE2F	rtcisE2F functions as a scaffold of IGF2BP2	m6A	Reader	IGF2BP2/YTHDF2	/	/	/	rtcisE2F-IGF2BP2/YTHDF2-E2F6/E2F3-Wnt/beta-catenin axis	[129]
hsa_circ_0062682	Hsa_circ_0062682 binds to YBX1	m5C	Reader	YBX1	Upregulated	Poor	/	Hsa_circ_0062682/YBX1 axis	[130]
circDLG1	circDLG1 sponges miR-141-3p to regulate the expression of WTAP	m6A	Writer	WTAP	Upregulated	Poor	Affect the susceptibility of HCC to sorafenib	circDLG1/miR-141-3p/WTAP axis	[131]
CircMAP2K4/miR-139-5p	circMAP2K4 binds with hsa-miR-139-5p to promote YTHDF1 expression	m6A	Reader	YTHDF1	Upregulated	Poor	/	circMAP2K4/hsa-miR-139-5p/YTHDF1 axis	[132]
CircGPR137B/miR-4739	circGPR137B sponges miR-4739 to upregulate its target FTO	m6A	Eraser	FTO	Downregulated	Favorable	Suppress cell growth	circGPR137B/miR-4739/FTO feedback loop	[133]

### MiRNAs regulate methylation-related proteins in HCC

MiRNAs can regulate oncogenes and tumor suppressors during the progression of liver cancer [134–136]. They are also implicated in liver cancer metastasis, immune modulation, and chemotherapy resistance, among other processes. Notably, miRNAs also regulate the expression levels of methylation-related proteins, thereby influencing the tumorigenesis of HCC through intricate mechanisms. WTAP is notably overexpressed in HCC tissues and exhibits a positive correlation with a poor prognosis in patients with HCC. Liu et al. found that miR-1395p attenuates WTAP expression by targeting its 3′-UTR, thereby inhibiting the growth of HCC [113]. The miR-139-5p/WTAP axis also governs HCC development by modulating EMT. miR-1275 directly targets IGF2BP1, IGF2BP2, and IGF2BP3. The overexpression of miR-1275 inhibits the expression levels of these IGF2BPs, thereby mitigating the malignant behavior of HCC [114]. In parallel, another study demonstrated that phytochemicals, including Tamarix articulata, quercetin, and epigallocatechin gallate, significantly increase the expression of miR-1275, thereby inhibiting the mRNA expression of IGF2BP1 and IGF2BP3 [115]. This suggests that the miRNA/IGF axis could serve as a novel mechanism for these phytochemicals to exert their anti-tumor effects. Zhou et al. found that miR-188-5p can bind to the 3′-UTR of hnRNPA2B1 and regulate the expression of hnRNPA2B1 [116]. Endoplasmic reticulum stress can facilitate sorafenib resistance in HCC through the miR-188-5p/hnRNPA2B1/PKM2 axis.

ALYREF is dysregulated in HCC, and its overexpression is closely linked to a poor prognosis in HCC. Xue et al. found that miR-4666a-5p and miR-6124 are potential regulators of ALYREF, suggesting their significant involvement in the epigenetic regulation of HCC [117]. Han et al. found that miR-3190 downregulates ALKBH5 expression in bone-metastasized HCC [118]. Reduced ALKBH5 levels facilitate HCC progression by regulating gene expression through both m6A-dependent and non-dependent pathways. TET2, crucial for hematopoietic stem cell self-renewal, exhibits low expression in HCC, inhibiting stemness and metastasis of HCC cells. Alcohol consumption further decreases the expression of TET2 in HCC. Further studies indicate that miR-22-3p directly targets TET2, and chronic alcohol intake instigates HCC tumor formation via the β-catenin/miR-22-3p/TET2 axis [119]. The expression of TET1 is decreased in HCC, which may have tumor suppressive effects. Lin et al. found that MiR-29b inhibits HCC metastasis by targeting TET1 [120]. Another study showed that miR-520b curbs HCC cell proliferation through the 3′UTR of TET1 mRNA [121]. These findings indicate the potential of targeting TET1 as a therapeutic strategy for the treatment of HCC.

### LncRNAs regulate methylation-related proteins in HCC

LncRNAs have also been reported to interact with methylation-related proteins, thereby influencing their functionality [137]. As expounded by Zhou et al., LINC00839 acts as a sponge for miR-144-3p, effectively downregulating the activity of miR-144-3p [122]. This, in turn, leads to elevated expression of WTAP, fostering the malignant phenotype within HCC cells. Bo et al. found that the lncRNA ILF3-AS1 can recruit METTL3, thereby elevating the m6A modification level of ILF3 [123]. In addition, the lncRNA ILF3-AS1 enhances the interaction between ILF3 mRNA and IGF2BP1, thereby contributing to the malignancy observed in HCC. A few methylation-related proteins are under the regulation of more than one miRNA or lncRNA. Studies have reported that miR503HG interacts with HNRNPA2B1, effectively curbing NF-κB signaling by modulating the ubiquitination status of HNRNPA2B1, consequently restraining HCC metastasis [124]. Moreover, lncRNA-UC002MBe-2 also engages with HNRNPA2B1, facilitating AKT inactivation and p21 induction [125]. This interaction plays a role in the suppressive impact of trecomycin on hepatoma cells. In addition, distinct lncRNAs exhibit varying effects on methylation-related proteins. In p53-deficient-HCC cells, Linc01612 mediates the ubiquitination and degradation of YBX1 through physical interaction with YBX1, thus exerting its biological functions [126]. In addition, Zhao et al. found that the IncRNA AWPPH binds to the YBX1 protein, subsequently enabling YBX1 to activate SNAIL1 or PIK3CA [127]. This activation, in turn, fosters the growth and metastasis of HCC.

### CircRNAs regulate methylation-related proteins in HCC

CircRNAs and methylation modifications play a significant role in the progression of HCC, with their underlying mechanisms extensively documented. circRERE is highly expressed in HCC, and its downregulation significantly increases the m6A levels of GBX2, thereby promoting the upregulation of the methyltransferase ZC3H13 [128]. This cascade expedites the proliferation and invasion of HCC cells. A recently identified rt-circRNA, rtcisE2F, is highly expressed in HCC and liver tumor-initiating cells (TICs). Chen et al. found that rtcisE2F regulates the interactions between IGF2BP2 and YTHDF2 with E2F6/E2F3 mRNAs [129]. Consequently, this triggers the self-renewal of TICs through the Wnt/β-catenin pathway, contributing to the onset and metastasis of HCC. The circRNA hsa_circ_0062682 is upregulated in HCC and recognized as a carcinogenic determinant. The YBX1 protein was identified to bind with circ_0062682. However, the precise downstream molecular mechanisms of this circ_0062682 and YBX1 interplay on HCC progression remain a subject warranting further exploration [130].

Under specific circumstances, circRNAs can act as sponges for miRNAs, thus further modulating the expression of downstream target genes. For instance, circDLG1, also upregulated in HCC, exhibits correlation to a poor prognosis. circDLG1 functions as a sponge for miR-141-3p, thereby regulating the expression of WTAP and inhibiting the progression of HCC [131]. Furthermore, Chi et al. constructed a regulatory network of circRNA-miRNA-m6A RNA methylation regulators and unveiled that circMAP2K4 interacts with hsa-miR-139-5p, promoting the expression of YTHDF1 and thereby facilitating HCC cell proliferation [132]. Another study revealed that the downregulation of circGPR137B or the upregulation of miR-4739 is correlated to poor prognosis in patients with HCC. CircGPR137B localizes with miR-4739 in the cytoplasm, where it functions as a sponge, up-regulating the expression of its target protein, FTO [133]. In turn, FTO mediates m6A demethylation of ciGPR137B and elevates its expression. This establishes a feedback loop comprising the circGPR137B/miR-4739/FTO axis, implicated in the development of HCC.

## Effect of interactions between ncRNAs and methylations in Tumor immune microenvironment (TIME) of HCC

The TIME of HCC is highly heterogeneous, posing a considerable challenge to liver cancer immunotherapy. Epigenetic modifications have been extensively studied in the context of HCC and exert substantial influence on the TIME. Therefore, we further summarized the effects of the interplay between ncRNA and methylation on the TIME in HCC (Table 3). This endeavor is geared towards offering novel avenues for the enhancement of HCC immunotherapy strategies.

**Table 3:** Effect of interactions between lncRNAs and methylations in TIME of HCC

Methylation type	Category	Methylation-related proteins	NcRNA	Interaction between ncRNAs and methylations	Involved immune cells	Molecular mechanisms	Refs
m6A	Writer	ZC3H13	miR-362-3p/miR-425-5p	MiR-362-3p/miR-425-5p binds to the ZC3H13 3′UTR	CD4 + T cells, macrophages, neutrophils, and dendritic cells	miR-362 3p/miR-425-5p/ZC3H13 involved in poor prognosis and tumor immune infiltration in HCC	[138]
m6A	Writer/reader/eraser	ZC3H13, KIAA1429, YTHDC2, HNRNPA2B, ALKBH5, YTHDC1, WTAP, METTL3, FTO, METTL13, RBM15, and YTHDF2	miR-142	hsa-miR142-5p regulates m6A regulators	Follicular helper T cells, activated NK cells, regulatory T cells, M0 macrophages, naïve B cells, resting memory CD4 + T cells, and M2 macrophages	m6A RNA methylation modulators may affect the prognosis through PD-L1 and immune cell infiltration in HCC patients	[139]
m6A	Writer	METTL14	circFUT8	Exosomal miR-628-5p inhibits METTL14 expression, and METTL14 promotes circFUT8 m6A modification and facilitates its nuclear export to the cytoplasm	M1 macrophages	Exosomal miR-628-5p/METTL14/circFUT8 axis	[140]
m6A	Reader	IGF2BP2	miR4458HG	miR4458HG binds to IGF2BP2 and facilitates IGF2BP2-mediated target mRNA stability	Tumorassociated macrophages	miR4458HG-IGF2BP2-HK2/SLC2A1 (GLUT1) axis	[141]
m6A	Writer	METTL14	lncRNA MIR155HG	Upregulation of METTL14 promotes the m6A methylation of MIR155HG	T cells	METTL14/MIR155HG/PD-L1 axis	[142]
m5C	Eraser	TET2	lncRNA MIR22HG	Repression of the lncRNA MIR22HG induces TET2	CD3 + T cells	miR-22/NANOG/CD133 axis	[143]
m6A	Writer	WTAP, IGF2BP3	circCCAR1	WTAP-mediated m6A modification enhances circCCAR1 stability by binding IGF2BP3, and circCCAR1sponges miR127-5p to upregulate its target WTAP	CD8 + T cells	circCCAR1/miR-127-5p/WTAP axis	[144]
m6A	Reader	YTHDF1	CircRHBDD1	circRHBDD1 recruits the m6A reader YTHDF1 to PIK3R1 mRNA	T cells	circRHBDD1/YTHDF1/PIK3R1 axis	[145]
m5C	Eraser	TET1	circTRIM33-12	circTRIM33–12 upregulates TET1 expression by sponging miR-191	/	circTRIM33–12/miR-191/TET1 axis, reduced expression of circTRIM33–12 in HCC cells increases immune evasion	[146]

### Effect of interactions between miRNAs and methylations in TIME of HCC

**Fig. 3:**
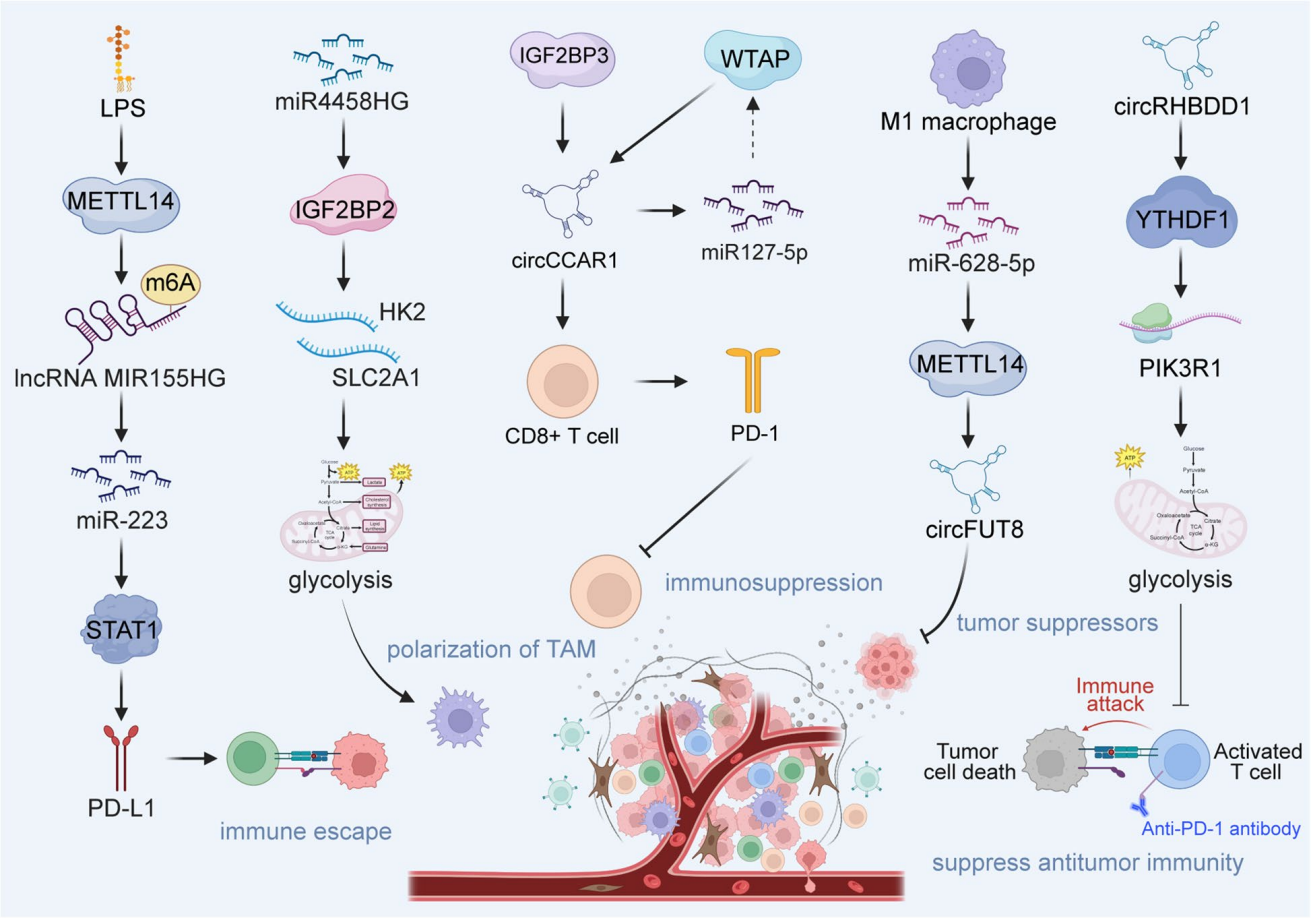
The effect of interactions between lncRNAs and methylations in tumor immune microenvironment of HCC. Created with BioRender.com

ZC3H13, an m6A writer, exhibits down-regulation in HCC tissues and could serve as a tool for evaluating the prognosis of patients with HCC. Wu et al. have identified miR-362-3p/miR-425-5p as upstream regulators of ZC3H13 capable of suppressing its expression. Subsequent investigations have revealed that ZC3H13 within HCC might contribute to disease progression by augmenting the infiltration of immune cells, specifically CD4^+^ T cells, macrophages, neutrophils, and dendritic cells, especially neutrophils [138]. In addition, ZC3H13 is closely linked to the expression of the immune checkpoint PD-L1, implying its involvement in regulating the immune microenvironment of HCC. Coincidentally, Lin et al. found that over 30 miRNAs contribute to the regulation of m6A methylation-related proteins, especially miR-142. Moreover, the m6A methylation of RNA affects the expression of PD-L1 and is closely linked to the infiltration of immune cells such as T follicular helper cells, activated NK cells, and regulatory T cells [139]. Among these, M1 macrophages stand out for their role in tumor suppression. Wang et al. demonstrated that M1 macrophage-derived exosomes transmit miR-628-5p to HCC cells, subsequently repressing the expression of METTL14 [140]. METTL14, in turn, facilitates the m6A modification of circFUT8, promoting its translocation from the nucleus to the cytoplasm, wherein M1 macrophages regulate the cirfut8/miR-552-3p/CHMP4B pathway, thereby inhibiting HCC progression (Fig. 3). miR4458HG is a carcinogen in HCC. Ye et al. found that miR4458HG binds to the m6A reader IGF2BP2, thereby enhancing the mRNA stability of IGF2BP2-mediated targets HK2 and SLC2A1 [141]. This alteration leads to a modification in the glycolytic response of HCC. In addition, HCC-derived miR4458HG exerts control over ARG1 expression and promotes the polarization of tumor-associated macrophages. Collectively, the intricate interplay of miRNAs and methylation may affect the progression of HCC by regulating the immune microenvironment.

### Effect of interactions between lncRNAs and methylations in TIME of HCC

The lipopolysaccharide (LPS) originating from intestinal bacteria can induce the upregulation of PD-L1 in HCC cells, thereby orchestrating T cell inhibition, thus affecting the development of HCC. Peng et al. revealed that LPS promotes the m6A methylation of the lncRNA MIR155HG by regulating the expression of METTL14, subsequently stabilizing the lncRNA MIR155HG [142]. MIR155HG regulates the expression of PD-L1 via the miR-223/STAT1 axis and assumes a key role in the immune evasion mechanisms within HCC. A pivotal challenge in effective cancer treatment lies in curtailing tumor recurrence subsequent to the expansion of tumor-initiating stem-like cells. Identifying effective combinations of TIC specificity is a promising strategy for prolonging survival in patients with HCC. Chen et al. demonstrated that the incorporation of immune checkpoint inhibitors yields an additional reduction in recurrence rates and extends the survival of patient-derived xenograft mice. Combination therapy with FDA-approved drugs that can inhibit the lncRNA MIR22HG reduces many toll-like receptors and stemness genes, and this downregulation induces PTEN and TET2, ultimately culminating in the loss of TIC self-renewal properties [143]. However, a more robust body of evidence is imperative to establish direct causality between lncRNA interactions and methylation and their collective influence on the immune microenvironment of HCC.

### Effects of interactions between circRNAs and methylation in TIME of HCC

Exosome-derived circRNAs can influence the immune escape of HCC by engaging in intercellular communication. Hu et al. showed that the level of circCCAR1 in exosomes from patients with HCC is increased [144]. WTAP facilitates the m6A modification of circCCAR1 by binding to IGF2BP3. As a consequence, circCCAR1 contributes to the impairment of CD8^+^ T cell function by stabilizing PD-1 protein, consequently fostering resistance to anti-PD-L1 immunotherapy. Another recently identified circRNA, circRHBDD1, was found to be highly expressed in patients with HCC who exhibited an anti-PD-1 response, which limited the effectiveness of anti-PD-1 therapy. Cai et al. found that circRHBDD1 recruits YTHDF1, thereby expediting the translation of PIK3R1 through m6A modification of PIK3R1 mRNA [145]. This, in turn, activates the PI3K/AKT signaling pathway, thereby promoting the progression of HCC. Inhibition of circRHBDD1 has been postulated to enhance the efficacy of anti-PD-1 therapy in immunodeficient mice. In addition, the downregulation of circTRIM33-12 in HCC is significantly associated with a poor prognosis. Mechanistically, circTRIM33-12 functions as a sponge for miR-191, thus facilitating heightened TET1 expression [146]. The consequent upregulation of TET1 operates in a manner that opposes oncogenic gene expression, inhibiting HCC proliferation, metastasis, and immune evasion. These findings illustrate the potential of circTRIM33-12 as a novel therapeutic target for HCC.

## RNA methylation-related proteins may serve as therapeutic targets for HCC

To date, it has been established that RNA methylation affects diverse aspects of HCC, such as its progression, immune microenvironment, and drug sensitivity. Most methylation-related proteins are dysregulated in HCC and play a key role in the development of HCC. Targeting methylation-related proteins holds great promise for the treatment of HCC.

### Targeting “writer” proteins for the treatment of HCC

Multiple studies have underscored METTL3’s potential as a therapeutic target in HCC, operating through diverse mechanisms. Silencing METTL3 has been shown to heighten HCC sensitivity to chemotherapy by impeding the m6A modification of p53 mRNA [147]. Furthermore, METTL3 fosters HCC metastasis by establishing a positive feedback loop with STAT3 [148]. Wang et al. have also reported that the METTL3 inhibitor STM2457 targets the epidermal growth factor receptor (EGFR) to improve the sensitivity of HCC to lenvatinib therapy [149]. This suggests that METTL3 might hold promise as a countermeasure against lenvatinib resistance in HCC. METTL14 targets EGFR, regulate the PI3K/AKT pathway, and inhibits the progression of HCC cells [150]. In addition, METTL14 may participate in the malignant development of HCC by mediating m6A methylation of cysteine sulfinic acid decarboxylase and glutamic-oxalotransaminase 2 [151]. Such findings suggest that targeted regulation of METTL14 could emerge as a novel avenue for the treatment of HCC. WTAP is significantly overexpressed in HCC, facilitating m6A modification that propels HCC progression via the ETS1-p21/p27 axis [152]. Therefore, inhibiting the expression of WTAP stands as a potential avenue for enhancing HCC prognosis. ZC3H13 may participate in transcriptional dysregulation or the JAK/STAT pathway in HCC [153]. Its expression is significantly associated with lymphocytes and immunomodulators. The upregulation of ZC3H13 inhibits the progression of HCC, thus designating it as a prospective therapeutic target for HCC.

### Targeting “reader” proteins for the treatment of HCC

HNRNPC plays a pivotal role in the development of HCC. Knocking down HNRNPC has been demonstrated to inhibit the proliferation, migration, and invasion of HCC cells via the Ras/MAPK signaling pathway [154]. Therefore, HNRNPC emerges as a potential therapeutic target for patients with HCC. YTHDF1 may enhance the malignant phenotype by promoting EMT and activating the AKT/glycogen synthase kinase-3 beta/beta-catenin signaling cascade [155]. Silencing YTHDF1 significantly inhibits the proliferation, invasion, and migration of HCC cells. In HCC, YTHDF2 may regulates the regulation of tumor-associated macrophage polarization, T cell dysfunction induction, and activation of T regulatory cells, thereby intricately influencing the course of HCC progression [156]. Hence, targeting either YTHDF1 or YTHDF2 presents a novel avenue for devising strategies for HCC treatment. A study showed that small nucleotide polymorphisms within YTHDC2 and FTO are significantly correlated to the prognosis of patients with HCC treated with TACE, suggesting that they may be potential targets to enhance treatment approaches for patients with unresectable HCC [157].

### Targeting “eraser” proteins for the treatment of HCC

FTO mediates IL-17 receptor A to regulate both inflammation and the transformation towards carcinogenesis in HCC [158]. Precisely targeting FTO may prevent HCC development, particularly in patients with hepatitis. Additionally, Xiao et al. reported that administering FTO-inhibiting nanomedicine to tumor-infiltrating dendritic cells proves advantageous in fostering HCC immunotherapy, especially when paired with immune checkpoint blockade post-HCC thermal ablation [159]. As demethylases, the ALKB family participates in the development of HCC. Research indicates that ALKBH1/2/3/4/7 is markedly highly expressed in HCC tissues and is correlated with the infiltration of immune cells such as CD8^+^ T cells, CD4^+^ T cells, and macrophages [160]. This suggests that the ALKB family may be a potential therapeutic target for HCC. Moreover, Chen et al. unveiled that ALKBH5 suppresses the expression of the oncoprotein LYPD1 in HCC in an M6A-dependent manner [106]. Furthermore, Qu et al. found that ALKBH5 deletion significantly inhibits the growth and migration of HBV-associated HCC cells [161]. HBx-ALKBH5 interactions might establish a positive feedback loop, contributing to the genesis of HBV-induced liver cancer. Consequently, targeting ALKBH5 surfaces as a promising avenue for managing HBV-induced HCC.

## Conclusions and perspectives

Accumulating evidence underscores the interplay between ncRNAs and methylation modifications in the development of HCC. On the one hand, RNA methylation modifications affect various facets of ncRNA, encompassing transcription, splicing, processing, translation, localization, and stability. These modifications affect biological processes such as proliferation, migration, invasion, apoptosis, and EMT in HCC. On the other hand, ncRNAs can also, in turn, regulate the expression levels of RNA methylation-related proteins, thereby exerting downstream molecular effects that shape the progression of HCC. In addition, the interaction between ncRNA and methylation-related proteins assumes a k ey role in orchestrating the immune microenvironment of HCC. Certain methylated modulators have shown potential to heighten HCC’s sensitivity to targeted therapies or chemotherapy and hold promise as viable therapeutic targets for HCC. However, existing research pertaining to the intricate interplay between ncRNAs and methylation modifications only scratches the surface. Future endeavors demand a more profound exploration to elucidate and validate the potential implications of ncRNA methylation modifications on HCC pathogenesis, consequently furnishing more precise strategies for HCC treatment.

## Data Availability

Data sharing is not applicable to this article as no new data were created or analysed in this study. Figures were created by Biorender (https://biorender.com/).

## Abbreviations

HCC: hepatocellular carcinoma
ncRNAs: non-coding RNAs
HBV: hepatitis B virus
HCV: hepatitis C virus
TACE: transarterial chemoembolization
miRNAs: microRNAs
lncRNAs: long ncRNAs
circRNAs: circular RNAs
mRNA: messenger RNA
UTR: 3′-untranslated region
tRNA: transfer RNA
m6A: N6-methyladenosine
m1A: N1-methyladenosine
m5C: 5-methylcytosine
Ψ: pseudouridine
m7G: 7-methylguanine
METTL3: methyltransferase-like 3
ALYREF: Aly/REF export factor
ZC3H13: zinc finger CCCH-type containing 13
ZCCHC4: zinc finger CCHC domain-containing protein 4
WTAP: Wilms tumor 1-associated protein
FTO: fat mass and obesity-associated protein
ALKBH5: alkylation repair homolog protein 5
YTHDF1: YTH N6-methyladenine RNA binding protein 1
IGF2BP1: insulin-like growth factor 2 mRNA-binding protein 1
HNRNPA2B1: heterogeneous nuclear ribonucleoprotein A2/B1
hnRNPC: heterogeneous nuclear ribonucleoprotein C
NSUN: NOL1/NOP2/SUN domain
DNMT2: DNA methyltransferase 2
TET: ten-eleven translocation
YBX1: Y-box binding protein 1
FSP1: ferroptosis suppressor protein 1
EMT: epithelial–mesenchymal transition
TIME: tumor immune microenvironment
LPS: lipopolysaccharide
EGFR: epidermal growth factor receptor
HDGF: hepatoma-derived growth factor
NML: nucleomethylin
TRMT6: tRNA methyltransferase 6
